# Exactitud del método de Cameriere y su variante, la Fórmula Europea, para la estimación de la edad en una población peruana subadulta

**DOI:** 10.21142/2523-2754-1003-2022-115

**Published:** 2022-09-28

**Authors:** Franz Yosset Bravo Molina

**Affiliations:** 1 Ministerio de Salud. Lima, Perú. franz.bravo.m@gmail.com Ministerio de Salud Lima Perú franz.bravo.m@gmail.com

**Keywords:** Odontología legal, Determinación de la Edad por los Dientes, Radiografía Panorámica, forensic dentistry, age determination by teeth, radiography, panoramic

## Abstract

**Objetivo:**

Determinar la exactitud del método de Cameriere y su variante, la Fórmula Europea, en una población peruana subadulta; y determinar una ecuación predictora de la edad cronológica.

**Materiales y métodos:**

Estudio descriptivo, observacional, transversal y retrospectivo. Se utilizaron 257 radiografías panorámicas digitales de pacientes con dentición mixta de 5 a 12 años, que se atienden en el centro odontológico Dental Científica, de la Universidad Científica del Sur, en la ciudad de Lima, Perú. Las variables morfológicas y el género de los sujetos fueron utilizados como variables predictoras para la estimación de la edad. Para evaluar la exactitud de cada método de estimación, se utilizó el error de predicción promedio y, para generar la ecuación predictora de la edad cronológica, se utilizó la regresión lineal múltiple.

**Resultados:**

Se encontró que el error promedio de predicción entre la edad cronológica con respecto a la edad estimada por la fórmula del método de Cameriere y la Fórmula Europea fue 0,73 ± 0,55 años y 0,77 ± 0,57 años respectivamente. Se generó un modelo cuyas variables predictoras de la edad fueron el *s* y *N0*, ambas variables fueron estadísticamente significativas y el R^2^ ajustado por el número de variables fue 75,96%.

**Conclusiones:**

Se determinó que la fórmula del método de Cameriere fue más exacta que la Fórmula Europea; sin embargo, no existió diferencia estadísticamente significativa entre ambas. Adicionalmente, se estableció una fórmula de regresión lineal específica para una población peruana de 5 a 12 años, con una adecuada capacidad de predicción.

## Abstract (en)

**Objective:**

To determine the accuracy of the Cameriere method and its variant, the European Formula, in a sub-adult Peruvian population; and determine a chronological age predictor equation.

**Materials and methods:**

Descriptive, observational, cross-sectional and retrospective study. It was used 257 digital panoramic radiographs of patients in mixed dentition from 5 to 12 years of age belonging to the "Dental Cientifica" Dental Center of the Universidad Cientifica del Sur in the city of Lima, Peru. All morphological variables and gender of the subjects were used as predictor variables for age estimation. To evaluate the accuracy of each estimation method, the age of each individual was compared with the estimated age using the mean prediction error. Multiple linear regression was used to generate the predictive equation for chronological age.

**Results:**

It was found that the mean prediction error between the chronological age with respect to the age estimated by the Cameriere method Formula and the European Formula was 0.73±0.55 years and 0.77±0.57 years, respectively. A model was generated whose predictor variables for age were *s* and *N0*, both variables were statistically significant and the Adjusted R^2^ for the number of variables was 75.96%.

**Conclusion:**

It was determined that Cameriere's Formula was more accurate than the European Formula; however, there was no statistically significant difference between the two. Additionally, it was possible to establish a specific linear regression formula for a Peruvian population aged 5 to 12 years with an adequate predictive capacity.

## INTRODUCCIÓN

La odontología forense se ha constituido como una herramienta fundamental en los procedimientos de identificación con fines médico-legales, tanto en sujetos vivos como en cadáveres. Entre las diversas áreas que manejan esta especialidad de la odontología, la estimación de la edad de una persona sin fecha de nacimiento comprobado es una práctica pericial forense común dentro de la investigación médico legal ^1^.

En niños, los métodos de estimación de edad que utilizan patrones dentales son considerados ágiles y altamente confiables, lo cual no solo es importante para la identificación de víctimas en casos de desastres masivos, sino también en al área médico-legal. Para ello, existen dos maneras: la primera, analizar la erupción y emergencia de las piezas dentales del peritado, y la segunda, evaluar radiográficamente la mineralización de las raíces y coronas de piezas deciduas y permanentes. Con base en lo anterior, se han desarrollado una gran variedad de métodos radiográficos que analizan los grados de mineralización a través de tablas y fórmulas ^2^.

Uno de estos métodos es el desarrollado por Cameriere *et al*. ^3^ quienes desarrollaron un nuevo concepto de estimación de la edad a través de la medida de ápices dentales abiertos. En su estudio, utilizó una muestra de 455 niños caucásicos italianos de 5 a 15 años, con el objetivo de presentar un método para la estimación de edad basado en la relación entre la edad y la medida de los ápices abiertos de piezas dentales permanentes mandibulares del lado izquierdo, utilizando radiografías panorámicas digitalizadas obteniendo una fórmula de regresión lineal. El estudio confirmó la validez de este método dental en la estimación de edad en niños ^3^.

A nivel mundial, diversos autores han aplicado el presente método o modificado el modelo original de regresión lineal en muestras de diversas nacionalidades. Cameriere *et al*. ^4^ realizaron un estudio para mejorar y ampliar la investigación original de Cameriere, con un mayor número de niños europeos de Croacia, Alemania, Kosovo, Italia, Eslovenia, España y Reino Unido, con el fin de crear una fórmula común útil para todos los países mencionados. Para el estudio se utilizaron 2652 radiografías panorámicas de niños caucásicos europeos entre las edades de 4 a 16 años, y se obtuvo una nueva fórmula de regresión lineal a la cual se denominó Fórmula Europea. 

En este sentido, el presente estudio presenta dos propósitos: el primero, determinar la exactitud del método de Cameriere y su variante, la Fórmula Europea, en una población peruana subadulta; y el segundo, determinar una ecuación predictora de la edad cronológica para niños peruanos de 5 a 12 años. 

## MATERIALES Y MÉTODOS

El estudio fue aprobado por el comité de ética de la Universidad Científica del Sur. Su diseño fue descriptivo, observacional, transversal y retrospectivo. La muestra estuvo conformada por 257 radiografías panorámicas digitales de pacientes con dentición mixta de 5 a 12 años obtenidas de la base de datos del centro odontológico Dental Científica, de la Universidad Científica del Sur, durante el periodo 2011-2017. Los criterios de inclusión fueron la buena calidad de las radiografías panorámicas digitales y los registros de edad en el rango establecido. Los criterios de exclusión fueron las radiografías panorámicas digitales donde se evidencien alteraciones dentales de forma, tamaño y número; así como la evidencia de tratamientos de ortodoncia. 

Se evaluaron las siete piezas dentales correspondientes al lado inferior izquierdo a través del *software* EasyDent V4 Simple Viewer. El número de dientes con desarrollo apical completo (*N0*) fue calculado. Adicionalmente, se evaluaron los siguientes parámetros:

• Medición *Ai*: se midió la distancia de las paredes internas radiculares en el lugar más apical que presentara formación radicular o ápice en desarrollo.
• Medición *Li*: se realizó la medición desde la imagen radiopaca de la corona hasta la extensión radicular.
• Valor *xi*: se obtuvo a través de la división entre la distancia de las paredes internas radiculares y la longitud dentaria, *xi= Ai/Li*.
• Valor *s*: se obtuvo a través de la sumatoria de los valores *xi*

Una vez obtenidas las mediciones, se procedió a completar los valores en la fórmula de Cameriere y la Fórmula Europea para obtener la edad estimada:

• Fórmula de Cameriere: 8,971+0,375*g*+1,631*x5* + 0,674.*N0*-1,034.*s*-0,176*s.N0*
• Fórmula Europea: 8,387+0,282*g*-1,692*x5* + 0,835*N0*-0,116.*s*-0,139*s*.*N0*

Donde g es una variable igual a 1 para varones y 0 para mujeres.

Todas las variables morfológicas y el género de los sujetos fueron ingresados a una hoja de cálculo de Excel para usarlos como variables predictoras para la estimación de la edad. 

En el presente estudio, la exactitud fue definida como qué tan cercana fue la edad estimada con respecto a la edad cronológica, medida como la diferencia entre la edad dental y la edad cronológica. Para evaluar la exactitud de cada método de estimación, la edad de cada individuo fue comparada con la edad estimada utilizando el error de predicción promedio.

Se utilizó el programa Stata 14.0 para el análisis estadístico de los datos numéricos y categóricos. Para el análisis descriptivo, se utilizaron medidas de resumen para variable categórica, tales como frecuencia y porcentaje. Para el análisis bivariado se utilizó la prueba de comparación de proporciones para evaluar la precisión de los métodos para estimar edad. Además, se usaron las curvas ROC para confirmar la precisión de ambos métodos y el nivel de significancia será del 5%.

Para generar la ecuación predictora de la edad cronológica, se utilizó la regresión lineal múltiple.

## RESULTADOS

Se realizó el análisis descriptivo para los datos obtenidos con la edad cronológica (EC), la edad estimada por la Fórmula del Método de Cameriere (ECA) y la edad estimada por la Fórmula Europea (EFE). El promedio de EC fue 8,09 años con una mediana de 8.02 años y valores mínimos y máximos de 5,01 y 12,90, respectivamente. En cuanto a ECA, se obtuvo una edad promedio de 8,33 años, con una mediana de 8,18 años y valores mínimos y máximos de 5,49 y 11,09 años, respectivamente. Con respecto a EFE, se obtuvo una edad promedio de 8,29 años, con una mediana de 8,12 años y valores mínimos y máximos de 6,01 y 11,05 años, respectivamente (Tabla 1).

**Tabla 1:** Análisis descriptivo de las variables edad cronológica, edad estimada y variables morfológicas en pacientes de 5 a 12 años

	Media	DE	ME	VA	Min	Máx
EC	8,09	1,82	8,02	3,31	5,01	12,90
ECA	8,33	1,36	8,18	1,85	5,49	11,09
EFE	8,29	1,30	8,12	1,70	6,01	11,05
x3	0,29	0,15	0,29	0,02	0,01	0,82
x4	0,38	0,19	0,36	0,04	0,01	0,92
x5	0,49	0,24	0,48	0,06	0,01	1,23
x7	0,76	0,38	0,81	0,14	0,08	1,85
s	2,14	1,11	2,04	1,24	0,19	5,48
N0	1,32	1,18	1,00	1,39	0,00	3,00

EC: Edad cronológica

ECA: Edad estimada por método de Cameriere

EFE: Edad estimada por la Fórmula Europea del método de Cameriere

Se realizó la estimación de la edad dental a través del método de Cameriere (ECA) y se obtuvo una edad estimada promedio de 8,33 años (±1,36), con un promedio de 8,40 años (±1,40) para el sexo masculino y de 8,27 años (±1.32) para el sexo femenino. De la misma manera, se realizó la estimación de la edad dental a través de la Fórmula Europea de Cameriere (EFE) y se halló, en promedio, una edad estimada de 8,29 años (±1,30), siendo el promedio de 8,34 años (±1,32) para el sexo masculino y 8,24 años (±1,28) para el sexo femenino (Tabla 2).

**Tabla 2:** Estimación de la edad a través de la Fórmula del Método de Cameriere y su variante la Fórmula Europea en pacientes de 5 a 12 años

	Sexo	Media	DE
EC	Todos	8,09	1,82
	Masculino	8,14	1,89
	Femenino	8,04	1,75
ECA	Todos	8,33	1,36
	Masculino	8,40	1,40
	Femenino	8,27	1,32
EFE	Todos	8,29	1,30
	Masculino	8,34	1,32
	Femenino	8,24	1,28

EC: Edad cronológica

ECA: Edad estimada por la Fórmula del Método de Cameriere

EFE: Edad estimada por la Fórmula Europea del Método de Cameriere

El error promedio de predicción entre la edad estimada por la fórmula del método de Cameriere y la edad cronológica fue 0,73±0,55 años, mientras que el error promedio de predicción entre la edad estimada por la variante europea del método de Cameriere y la edad cronológica fue 0,77±0,57 años. Aunque la fórmula del método de Cameriere fue más exacta que la Fórmula Europea, no hubo diferencia estadísticamente significativa (p = 0,470). El error promedio de predicción entre la edad estimada por la fórmula del método de Cameriere y la edad cronológica fue 0,72±0,58 años en hombres y 0,74±0,53 años en mujeres. Se usó la prueba U de Mann Whitney para comparar ambos grupos y no se encontró diferencia estadísticamente significativa (p = 0,462). El error promedio de predicción entre la edad estimada por la variante europea del método de Cameriere y la edad cronológica fue 0,76±0,59 años en hombres y 0,77±0,56 años en mujeres. Se usó la prueba U de Mann Whitney para comparar ambos grupos y no se encontró diferencia estadísticamente significativa (p = 0,709) (Tabla 3).

**Tabla 3:** Comparación entre la exactitud de las edades estimadas a través del Método de Cameriere y su variante la Fórmula Europea de Cameriere en pacientes de 5 a 12 años

	Sexo	ED-EC	EP	DE	Me
| ECA - EC |	Total	0,24	0,73	0,55	0,60
	Femenino	0,23	0,74	0,53	0,67
	Masculino	0,26	0,72	0,58	0,55
| EFE- EC |	Total	0,20	0,77	0,57	0,66
	Femenino	0,20	0,77	0,56	0,69
	Masculino	0,20	0,76	0,59	0,61

ED: Edad dental

EC: Edad cronológica

EP: Error promedio de predicción

ECA: Edad estimada por la Fórmula del Método de Cameriere

EFE: Edad estimada por la Fórmula Europea del Método de Cameriere

**Figura 1:**
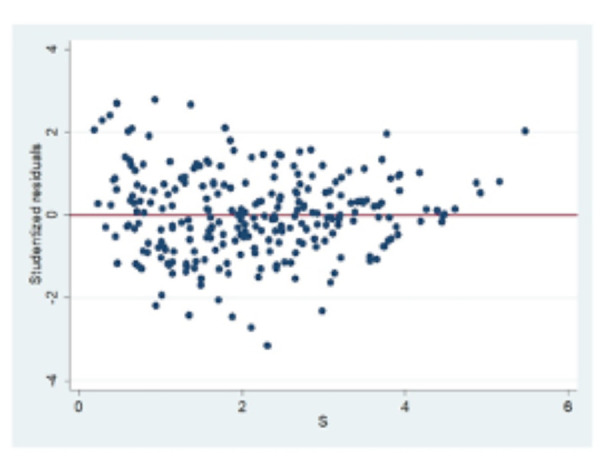
Gráfico para la evaluación de la linealidad entre los residuos estudentizados y la variable predictora (s), se observa un patrón aleatorio de los puntos.

Se evaluó la normalidad de los residuos estudentizados, a través de la cual se decidió utilizar un modelo de regresión lineal múltiple para la obtener una ecuación predictora de la edad cronológica. Este modelo determinó los factores predictores de la edad de los pacientes de 5 a 12 años. Las variables independientes fueron *x3*, *x4*, *x5*, *x7*, *s* y *N0*. Se evaluaron varios modelos de regresión lineal múltiple en función al R^2^ ajustado, lo cual explica la variabilidad de la variable edad real explicada por las variables predictoras ajustadas por el número de variables. Se generó un modelo cuyas variables predictoras de la edad fueron el *s* y el *N0*, las cuales fueron estadísticamente significativas, y el R^2^ ajustado por el número de variables fue del 75,96%. Los errores estándar eran pequeños, lo cual nos asegura la precisión de los coeficientes de regresión (Tabla 4). Se pudo determinar que la variable morfológica *s*, utilizada en la ecuación predictora, mostró un patrón de dispersión aleatorio en relación con los residuos estudentizados, lo cual determinó una correcta linealidad (Figura 1).

**Tabla 4:** Determinación de una ecuación predictora de la edad cronológica en pacientes de 5 a 12 años

	Coeficiente	Error estándar	p	IC (95%)
s	-0,988	0,089	p < 0,001	-1,163; -0,813
N0	0,47	0,0839	p < 0,001	0,305; 0,635
Constante	9,58	0,294	p < 0,001	9,001; 10,159

Regresión lineal múltiple, modelo estadísticamente significativo (p < 0,001), R2 ajustado: 75,96%

Edad estimada= 9,580 - 0,988(s) + 0,470(N0)

Se generó la siguiente ecuación predictora: 

Edad estimada = 9,580 - 0,988(s) + 0,470(N0) 

## DISCUSIÓN

La estimación de la edad biológica de un individuo se basa en los cambios morfológicos que suceden en ciertos tejidos, como los huesos y las piezas dentales. Estos cambios están determinados por el grado maduración y crecimiento fisiológico a lo largo de su vida. Los métodos de estimación de edad se basan en indicadores de la edad, los cuales, según su desarrollo, son utilizados como estimadores ^5^.

La estimación de edad en el contexto forense es de vital importancia en la legislación criminal y administrativa. La aplicación de métodos de estimación de edad representa una importancia invaluable en casos de relevancia médico legal como secuestros, abuso sexual, así como problemas judiciales en casos de empleo, mayoría de edad, beneficios sociales y maritales ^6^.

Existen diferentes métodos para la estimación de la edad como los de Demirjian, Nolla, Willems y Haavikko. Estos métodos, desarrollados para individuos en crecimiento, requieren del análisis radiográfico para evidenciar al desarrollo dental. Sin embargo, todos se remontan a algunas décadas, lo cual genera la necesidad de contar con nuevos métodos de estimación, considerando el cambio en la tendencia de crecimiento actual ^7^.

En el Perú, en la última década, se han realizado estudios sobre la estimación de la edad utilizando el método de Demirjian ^8^^-^^12^, el método de Cameriere ^13^, así como comparaciones realizadas entre los métodos de Demirjian y Ubelaker ^14^, Nolla y Demirjian ^(15, 16)^ y Moorees y Demirjian ^17^. Sin embargo, los resultados de estas investigaciones no terminan de ser concluyentes para el objetivo de determinar el método más preciso para estimar la edad en una población peruana en dentición mixta. 

En el contexto nacional, se evidencian casos de delitos contra la libertad sexual en menores de edad, maltrato infantil y delitos agravados, como el robo y sicariato, en los que los menores de edad son utilizados por personas que infringen la ley. Por lo tanto, estimar la edad en menores de edad es parte fundamental de la evaluación médico-legal y más aún cuando el código penal peruano establece que la edad del menor determina la magnitud de la pena recibida por el agresor ^18^.

Con base en lo mencionado, se decidió utilizar el método de Cameriere, debido a que la evaluación del proceso fisiológico de la maduración dental es uno de los sistemas que presenta mejores atributos en cuanto a la estimación ^19^. En 2006, Cameriere *et al*. ^3^ desarrollaron un método para estimar la edad cronológica en base a la relación entre la edad y la medida de los ápices abiertos de siete dientes mandibulares del lado izquierdo a excepción de la tercera molar. Este método ha sido aplicado en distintas poblaciones de diversas nacionalidades. Fonseca *et al*. ^20^ realizaron una revisión sistemática a través de cual comprobaron la aplicabilidad del método de Cameriere en poblaciones latinoamericanas (Brasil, México y Perú), con resultados alentadores.

En el presente estudio, se determinó que la edad estimada a través de la Fórmula del Método de Cameriere (ECA) sobrestimó la edad cronológica (EC) en 0,24 años; de la misma manera, se obtuvo una sobrestimación tanto para el sexo masculino (0,26 años) como para el femenino (0,23 años). El error de predicción promedio entre ECA y EC fue de 0,73± 0,55 años (varones = 0,72±0,58, mujeres = 0,74±0,53), lo que indica una exactitud similar para estimar la edad tanto en varones como en mujeres. Sin embargo, estudios como el de Cameriere *et al*. ^21^ determinaron un error de predicción de 0,75 años, con una ligera tendencia a la subestimación de en niños peruanos de 9,5 a 16,5 años. Wolf *et al*. ^22^ determinaron que el método de Cameriere presentó una alta inexactitud en una población alemana de 6 a 14 años, con una diferencia promedio de 0,07 en niños con sobrestimaciones en las edades de 6 a 11 años y subestimaciones en las edades de 12 a 14 años. En el caso de las niñas, se determinó una diferencia promedio de 0,08, así como sobrestimaciones en las edades de 6 a 10 años y subestimaciones en las edades de 11 a 14 años. Apaydin *et al*. ^23^ determinaron que el método de Cameriere subestimó la edad con un error de predicción promedio de en 0,580 (M = 0,603, F = 0,550) en niños turcos de 5 a 15,9 años. Pongo da Luz *et al*. ^24^ determinaron una subestimación en niños brasileños y croatas de -1,05 y -1,19 años, respectivamente, con una diferencia promedio absoluta de 1,26 y 1,38 para niños brasileños y croatas, respectivamente. No obstante, cuando la muestra fue estratificada por sexo y edad, el método de Cameriere presentó una alta exactitud en las edades más tempranas. Ozveren *et al*. ^25^ establecieron una subestimación (F = 0,37, M = 0,18) de la edad a través del método de Cameriere en niños turcos de 6 a 15 años, con una exactitud en la estimación de edad del 84,6% en niños y el 77,3% en niñas.

En 2007, Cameriere *et al*. ^4^ realizaron una investigación con el objetivo de mejorar y expandir la investigación realizada en el 2006, a partir de la relación entre la edad y la medida de los ápices abiertos, con un número mayor de niños de varios países europeos como Croacia, Alemania, Kosovo, Italia, Eslovenia, España y Reino Unido. A través del análisis de las variables morfológicas con respecto a la edad cronológica, describieron una fórmula de regresión lineal a la que se denominó Fórmula Europea.

En el presente estudio, se determinó que la edad estimada por la Fórmula Europea (EFE) sobrestimó la edad cronológica (EC) en 0,20 años, obteniendo una sobrestimación similar tanto para el sexo masculino como femenino (0,20 respectivamente). El error promedio de predicción entre EFE y EC fue de 0,77±0,57 años (varones = 0,76±0,59, mujeres = 0,77±0,56), lo que indica una similar exactitud para estimar la edad tanto en varones como en mujeres. Al respecto, De Luca *et al*. ^26^ determinaron la exactitud de la Fórmula Europea de Cameriere en una población mexicana de niños de 5 a 15 años, a través de la diferencia entre la edad cronológica con respecto a la edad estimada, y hallaron una ligera sobreestimación en niñas (0,10 años), pero una correcta estimación en niños, con una precisión de 0.00 y con un error de predicción de 0,63 para el sexo femenino y 0,52 para el masculino. Por el contrario, Balla *et al*. ^6^ compararon la Fórmula Europea de Cameriere con los métodos de Willems y Acharya en una población de niños del sur de India de 7 a 15 años y determinaron que la Fórmula Europea obtuvo una subestimación de -0,600 (F = -0,700, M = -0,510), con un error de predicción promedio de 0,580 (F = 0,540, M = 0,628), siendo mejor que los otros métodos. Rivera *et al*. ^27^ demostraron una alta exactitud y fiabilidad de la fórmula europea de Cameriere en la estimación de edad de niños colombianos de 6 a 14 años, con una sobreestimación de 0,08±0,68 en niños y subestimación en niñas de 0,25±0,65, y con un error promedio de predicción de 0,57±0,41 para el sexo femenino y 0,57±0,38 para el sexo masculino.

En el presente estudio, al comparar la exactitud de la Fórmula del Método de Cameriere con respecto a su variante, la Fórmula Europea, se pudo determinar que la primera más exacta; sin embargo, no existió una diferencia significativa, por lo que ambos métodos pueden ser utilizados en la estimación de la edad en una población peruana de 5 a 12 años.

Con respecto a la determinación de una fórmula de regresión lineal múltiple, se encontró que no todas las variables utilizadas por la fórmula del método de Cameriere ^3^ y la Fórmula Europea ^4^ fueron estadísticamente significativas, siendo el R^2^ ajustado del 83,6% y el 86,1%, respectivamente, en ambos estudios. En esta investigación se estableció que las variables número de ápices cerrados (*N0*) y la sumatoria de los ápices abiertos normalizados (*s*) contribuyeron significativamente a la nueva fórmula (p < 0,001) y se obtuvo un R^2^ ajustado del 75,96%, lo cual determina que las variables mencionadas presentaron una elevada capacidad de predicción de la edad en una población subadulta peruana en un rango de edad de 5 a 12 años. 

Al respecto, Attiguppe *et al*. ^28^ establecieron una fórmula específica para niños de la India (Davangere) de 6 a 15 años, en la cual las variables que se utilizaron fueron *N0* y X4, y se encontró un R^2^ ajustado del 73,5%, lo cual indica que se debe prestar atención a la posible diferencia entre los niños de diferentes orígenes étnicos. De la misma manera, Sharma *et al*. ^29^ determinaron que el género además de las variables X4, X1, X6, s, N0 contribuyeron significativamente en el análisis de regresión lineal en niños de 5 a 15 años del norte de India, y se obtuvo una fórmula especifica con un R^2^ de 85,5%. Haliah *et al*. ^30^ establecieron una fórmula específica en niños del norte de Alemania de 5 a 16 años en la cual las variables de sexo (g), la suma de los ápices abiertos normalizados (s), el número de ápices cerrados (*N0*) y el primer orden de interacción entre el factor X3 y *N0* contribuyeron significativamente a la fórmula, lo que explica un 84,1% del total de la desviación, con una edad media de 0,070 años y 1,185 años del rango intercuartil (IQR). Mazzilli *et al*. ^31^ establecieron un análisis de regresión lineal modelado para una población brasileña de niños de 4 a 16 años en cuyo modelo los predictores de edad de sexo, X5, N0, S y S*N0 fueron estadísticamente significativos encontrando un R^2^ de 91,2% determinando que dicha fórmula no subestima o sobreestima la edad en ambos sexos. Gannepalli *et al*. ^32^ analizaron una muestra de niños de 10 a 15 años y propone un análisis de regresión que demostró que no todas las variables usadas por Cameriere en su modelo europeo fueron significativos como predictores de la edad en una muestra del sur de India, el estudio reveló que las variables de intercepción y sumatoria de los ápices abiertos (*s*) fueron predictores de la edad y explican un 88,3% del total de la desviación (R^2^ 0.883).

## CONCLUSIONES

Se pudo determinar que la fórmula del método de Cameriere fue más exacta que la Fórmula Europea; sin embargo, no existió diferencia estadísticamente significativa entre ambas, y se puede utilizar adecuadamente ambos métodos para la estimación de la edad en niños peruanos de 5 a 12 años. De la misma manera se pudo establecer una fórmula de regresión lineal específica para una población peruana de 5 a 12 años con una adecuada capacidad de predicción.

## Financiación:

Autofinanciado
